# Influence of a Zombie-like State of the Liver on Drugs and Its Medico-Legal Implications: A Scoping Review

**DOI:** 10.3390/ph18060787

**Published:** 2025-05-24

**Authors:** Ivan Šoša

**Affiliations:** Department of Anatomy, Faculty of Medicine, University of Rijeka, 51000 Rijeka, Croatia; ivan.sosa@uniri.hr

**Keywords:** hepatocyte senescence, hepatocyte senescence-associated secretory phenotype (hepatocyte SASP), medico-legal, pharmaceuticals, substances of abuse

## Abstract

When cells remain permanently trapped in a particular cell cycle stage, they are in senescence. This also occurs in the liver. Such cells are often referred to as “zombie cells”, and an entire organ filled with these “zombie cells” is said to be in a “zombie-like” state, characterized by a lack of function. The senescence-associated secretory phenotype (SASP) encompasses the substances these “zombie cells” release, which can significantly affect nearby cells and tissues. While cellular senescence and SASP are related concepts, they are distinct. This scoping review aims to clarify the role of hepatocyte senescence and hepatocyte SASP in the administration of pharmaceuticals, as well as their relevance to medico-legal practice, disability claims, and insurance coverage. In this context, the effects of pharmaceuticals on senescent hepatocytes are discussed, particularly regarding the medico-legal implications of substances likely to be abused. In conclusion, hepatocyte senescence may be relevant in clinical or medico-legal work because it sheds a new light on interpreting clinical findings and expert witness statements.

## 1. Zombie-like State

There are four key signs of cellular senescence in hepatocytes: (1) permanent cell cycle arrest, (2) disrupted bioenergetics, (3) resistance to apoptosis, and (4) the secretion of pro-inflammatory cytokines [1,2,3]. Specifically, senescent hepatocytes undergo morphological changes, including flattened cell bodies, vacuolization and granularity in the cytoplasm and abnormal organelles, and increased nuclear polyploidy [4,5,6]. These alterations are also associated with lipid and glucose uptake [7,8]. Likewise, senescent hepatocytes tend to cluster together [9]. Senescent hepatocytes may be identified using classical senescence markers, including p21, heterochromatin protein 1β, and senescence-associated β-galactosidase activity; they also show decreased levels of the proliferation marker Ki67 [10,11,12].

Cells trapped in permanent cell cycle arrest are called “zombie cells” [13]. An entire organ with “zombie cells” that have entered a state characterized by a lack of function is referred to as being in a “zombie-like” state [14]. This condition can lead to various detrimental outcomes, including the progression of liver disease. In younger organisms, the immune system effectively responds to and eliminates senescent cells [15,16].

### 1.1. Inflammation and Senescent Hepatocytes

Inflammation is an essential aspect of the body’s response to injuries. It usually plays a significant role in tissue healing and restoring homeostasis. [17,18]. Insight into this process, specifically examining wounds, is essential in medico-legal practice. This insight is, to its essence, a tool for clarifying the relationship between the causes and mechanisms of the injury on one hand, and the wounds present on the other [19]. The process in the background is complex, dynamic, and consists of four distinct phases [6]. The first phase manages the bleeding, minimizes blood loss, and activates clotting factors. Cytokines such as tumor necrosis factor-alpha (TNF-α), interleukin-1 (IL-1), and IL-6 are released to enhance clot formation. Hemostasis, the management of bleeding, is followed by the inflammation phase. During this phase, cytokines such as TNF-α and transforming growth factor-beta (TGF-β) promote the chemotaxis of pro-inflammatory mediators to the wound site (including neutrophils, macrophages, and fibroblasts). These cells work to clear cellular debris and pathogens. Likewise, the stimulation of inflammatory macrophage migration has been documented due to the ability of the senescent hepatocyte-conditioned medium to support cellular culture. However, this same medium has no influence on the chemotaxis of non-inflammatory ones, potentially contributing to a pro-inflammatory microenvironment in vivo, or aiding in the clearance of senescent cells [11,20]. After the inflammation phase, the proliferation phase starts, during which anti-inflammatory cytokines such as IL-4, IL-10, and IL-13 are released, stimulating the formation of granulation tissue and inhibiting the further release of inflammatory mediators to facilitate tissue repair. Finally, IL-4, IL-10, and IL-22 contribute to the reorganization of collagen fibers, increasing the strength of scar tissue and enhancing wound integrity.

Nevertheless, inflammation that occurs without an injury is unnecessary and can harm a healthy organism.

### 1.2. Hepatocyte Senescence vs. Hepatocyte Senescence-Associated Secretory Phenotypes

Generally, cellular senescence is a state where cells permanently stop dividing. The SASP refers to the substances these senescent cells release, which can significantly impact nearby cells and tissues [1,21]. These are two related but distinct concepts. Cells cease to divide while in cellular senescence, but stay viable and energetically active. They show alterations in morphology, gene expression, and epigenetic markers, including heightened DNA damage, telomere shortening, and epigenetic alterations [22,23,24]. Consequently, they can act as a tumor suppressor mechanism, preventing damaged cells from proliferating and potentially becoming cancerous [23,24,25]. Senescent cells may evoke an anti-tumor immune response in an organism’s fight against cancer [25], so-called “senescence surveillance”—mediated by the cytokines within SASP. This has been shown to suppress the progression of malignancy in hepatocytes in a mouse model. Other mechanisms of tumor suppression have been proposed [24,26,27].

On the other hand, the senescence-associated secretory phenotype (SASP) refers to the secretome of senescent cells, composed of various bioactive molecules that can promote inflammation, tissue damage, and remodeling [28,29]. These molecules, including cytokines, chemokines, growth factors, and proteases, can also influence the behavior of neighboring cells. While the SASP can provide beneficial effects, including aiding in wound healing and tissue repair, it can also contribute to chronic inflammation, cancer, and aging.

This scoping review aims to clarify the role of hepatocyte senescence and hepatocyte SASPs in pharmaceutical administration and their relevance to medico-legal practice.

## 2. Hepatocyte Senescence-Associated Secretory Phenotype and Tissue Remodeling

Wound healing is a physiological process guided by cellular senescence. It involves tissue repair after injury and comprises three main stages: inflammation, tissue formation, and remodeling [30].

The SASP has been thoroughly studied across various cell types, revealing that different cell types exhibit distinct yet overlapping characteristics [3,20,27]. Cellular senescence plays beneficial roles in embryonic development, wound healing, resolving fibrosis, and tumor suppression [5]. It is believed to be evolutionarily attained as an antitumor mechanism where the SASP recruits immune cells to facilitate senescent cell removal [31]. The basis for its harmful effects is not fully understood. However, senescent cells can alter their microenvironment by adopting specific yet negative ‘secretory phenotypes’ that include cytokines, chemokines, growth factors, and proteases [5]. The primary function of these phenotypes is to exert a pro-inflammatory effect on nearby cells. One of the main earmarks of cellular senescence used in wound healing is cell cycle arrest. It results in the elimination of damaged cells by macrophages [20,32].

Current evidence suggests that transient cellular senescence can aid tissue repair; however, the prolonged presence of senescent cells may hinder this process. To illustrate the overlapping characteristics of SASP in various cell types, we aim to explore skin wound healing. A crucial molecule in this process is the matricellular protein CCN1, which is primarily sourced from hepatocytes [33]. This molecule can induce senescence in fibroblasts or myofibroblasts, which helps reduce fibrosis. In corneal wound healing, senescent fibroblasts exert reduced responses to fibroblast growth factor 2 (FGF2, also known as basic FGF) [34,35]. Senescent fibroblasts display an anti-fibrogenic phenotype marked by the presence of platelet-derived growth factor BB and an elevated expression of matrix metalloproteinase (MMP) -1, -3, and -13 [36,37,38,39]. In the nervous system, the wound healing activity of astrocytes was found to be impaired by induced senescence in a Tenovin-1 treatment experiment [40,41]. Hepatocyte SASPs can contribute to cardiovascular disease (CVD) development via inflammation, oxidative stress, and endothelial dysfunction. They can also contribute to vascular remodeling by increasing vascular stiffness and reducing vascular compliance [42]. The same oxidative stress triggers caveolin 1–PTRF signaling in diabetes, leading to cellular senescence through the p53–p21 pathway [5,43]. This diabetes-induced senescence, along with a CXCR2-enriched SASP, hinders wound healing [2]. Fibroblasts in the lung induce the G2/M cell cycle arrest of alveolar epithelial cells, leading to the aberrant repair of tissue damage and re-epithelialization [44,45]. Senescent mesenchymal stem cell (MSC)-derived extracellular vesicles also inhibit wound healing via a mechanism involving the downregulation of miR-146a [46]. An upregulated microRNA in senescent hepatocytes promotes inflammation and tissue damage [12]. Another microRNA, miR-29a, is downregulated in senescent hepatocytes, impairing liver function and fibrosis [47,48].

Inflammation-mediated cellular senescence reduces fibroblast proliferation and migration, disrupting wound healing. These processes are essential for new tissue formation [2,49]. Otherwise, DNA damage response (DDR) and reactive oxygen species (ROS)–p16 signaling steer this process elsewhere [50].

## 3. Scoping Review

### 3.1. Methodology

The present literature review aimed to address the following question: Can this review identify hepatocyte senescence or the hepatocyte SASP relevant to the medico-legal interpretation of injury? The query terms were checked using the Open Science Framework (OSF) to determine whether this research was eligible, ensuring a comprehensive review without overlapping with existing reviews. This process helped determine whether a scoping review on the topic had already been conducted, and if there was enough literature to justify such a review.

The Google Scholar database was searched for literature on 7 April 2025 including all papers since its inception. A third-party software (Zotero 7.0.15 [Windows 11], Corporation for Digital Scholarship. https://www.zotero.org/ accessed on 23 April 2025) was used to transfer the search results from the browser to the reference management tool. The protocol for this search was also registered with the OSF, and the DOI number 10.17605/OSF.IO/2GYZC was assigned to that project [51].

**Figure 1:**
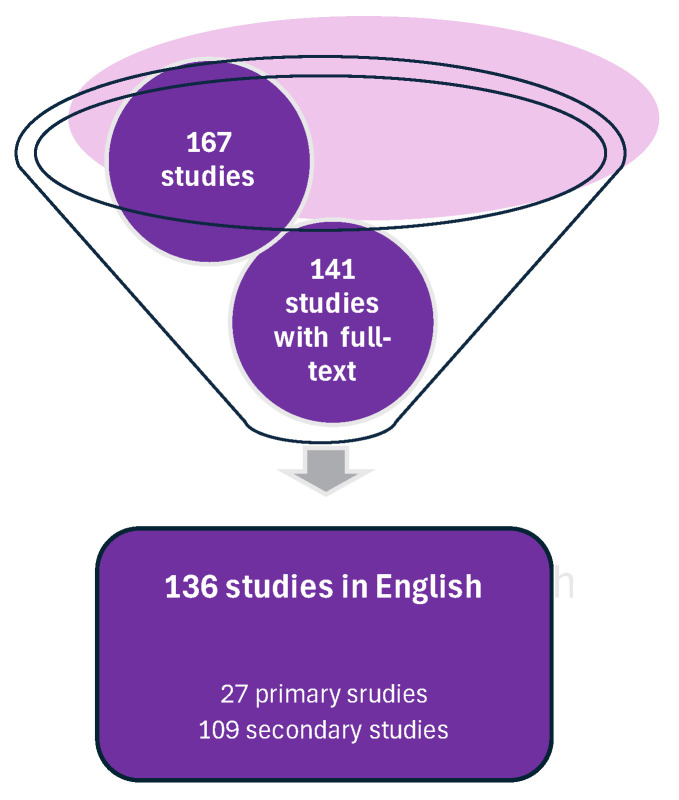
Search strategy identified 167 studies, 27 of which were primary studies. This strategy was based on the guidelines from the Preferred Reporting Items for Systematic reviews and Meta-Analyses (PRISMA) version PRISMA 2020.

The search for “hepatocyte” AND “cellular senescence” AND “forensic/medicolegal” in any field yielded 178 results, confirming that a scoping review is an appropriate method. After removing 11 duplicates, the final search resulted in a total of 167 studies (Figure 1).

### 3.2. Primary Studies

This review identified three conference papers, eight doctoral theses, six case reports, and ten research papers in the primary studies. Out of 27 studies, 7 (25.9%) involved human subjects, while 8 (29.6%) were based on animal models. The remaining six studies (22.2%) were laboratory studies that involved in vitro experiments and cell culturing.

### 3.3. Secondary Publications and Non-Peer-Reviewed Material

After thoroughly examining the remaining 109 secondary publications and non-peer-reviewed materials, this review identified 1 lecture (0.92%), 2 master’s dissertations (1.83%), 21 books or book sections (19.27%), and 85 journal articles (78%). Among these, only four journal articles included a “risk of bias” assessment (3.67%), while a specialized framework considering the population, intervention, comparison, and outcome (PICO) was displayed in tabular format in 21 publications (19.27%).

## 4. Clinical Implications

Senescence of all types of cells in the liver, including hepatocyte senescence and hepatocyte SASPs, is gaining recognition not only for its role in chronic liver diseases, but also for its potential implications in the medico-legal sphere [3,6]. Hence, senescence directs increased inflammation and oxidative stress, which can damage blood vessels, and increase vascular permeability and extravasation [52,53]. Another mechanism of impairing vascular integrity is reducing the expression of vascular endothelial cadherin, a key component of intercellular junctions [54]. Alterations in the cellular microenvironment and SASP-associated cytokines, such as IL-1β and TNF-α, can increase vascular permeability, allowing blood to leak into tissues and cause bruising, otherwise affecting the normal healing process [20,55,56].

Stakeholders such as insurance companies can benefit from understanding the role of liver senescence or hepatocyte SASPs, as well as the significant impairment of related liver function [57]. As important as this might be in disability claims and insurance coverage, any case of violence/injury should bear in mind the potential impact of senescent livers [3,58]. This understanding can help companies accurately assess risk, determine coverage, and calculate damages. It can inform decisions related to settlement amounts and support for rehabilitation [59,60]. A better understanding of hepatocyte senescence can also guide regulatory decisions regarding pharmaceuticals, chemicals, and environmental toxins that may impact liver health [61]. Nevertheless, leveling up the knowledge of medical professionals—in general, regarding hepatocyte senescence and hepatocyte SASPs—can help develop more effective treatment strategies for patients who have experienced trauma.

By understanding the complex interplay between hepatocyte senescence, hepatocyte SASPs, and injuries, stakeholders such as expert witnesses and medico-legal pathologists can deliver more informed opinions (expertise) on the severity of injuries [6,27]. Quantifying the level of cellular senescence directly in a medico-legal setting can be challenging [62,63]. Still, it is relevant since senescent livers often show impaired function, including reduced activity of cytochrome P450 enzymes (Phase I metabolism) [64] and conjugating enzymes (Phase II metabolism) [65].

Knowledge of hepatocyte senescence and hepatocyte SASPs may be useful in discussing an individual’s ability to make informed decisions and capacity to take actions, potential long-term consequences, and the impact of pre-existing liver conditions [66]. The absence of secondary publications thematically linked to the “expert witness” (Table 1) may stem from expert witnesses using comprehensive knowledge from their expertise, or there may be too many pronounced biases to warrant publications on the topic. In addition to providing valuable knowledge about hepatocyte senescence to inform opinions on the severity of injuries, examining liver tissue from victims may have significant medicolegal implications in toxicology, pharmaceutical, and toxic injury litigation and transplantation [14].

Understanding the relevance of hepatocyte senescence and hepatocyte SASPs in this context of legal medicine can potentially improve patient outcomes and reduce long-term complications for injured individuals [57,67,68].

## 5. Pharmaceuticals and Senescent Hepatocytes

Pharmaceutical metabolism in senescent hepatocytes can change due to alterations in gene expression, enzyme activity, and overall cellular function [3,7,9]. Aging hepatocytes demonstrate the modified expression of transporters involved in the uptake and efflux of compounds, such as OATP1B1 and P-glycoprotein (P-gp) [69,70]. There is decreased expression of cytochrome P450 (CYP) enzymes, including CYP3A4, CYP2C9, and CYP2D6, which are critical for the metabolism of many drugs [71]. This reduction in CYP enzyme activity leads to a decreased metabolism of pharmaceuticals, and hence to higher drug concentrations in the body, increasing the risk of adverse drug reactions and toxicity [72,73]. Senescent hepatocytes exhibit increased glucuronidation activity, which can also impact drug metabolism [7,9]. Changes in sulfation and methylation activities further impact the metabolism of pharmaceuticals [74]. Concurrently, increased oxidative stress, the heightened expression of inflammatory genes, or the reduced cellular uptake of pharmaceuticals, which have all been noticed in aging cells, can affect drug metabolism as well [75,76,77].

Psychopharmacological agents can affect hepatocyte function and may lead to senescence. Selective serotonin reuptake inhibitors (SSRIs) and tricyclic antidepressants (TCAs) have been linked to hepatotoxicity and may promote hepatocyte senescence [1,2]. Similarly, certain antipsychotics, such as olanzapine and quetiapine, are also associated with hepatotoxicity, and could contribute to hepatocyte senescence [3]. As a result of senescent changes, the metabolisms of several medications are affected. There is a reduction in the metabolism of statins, which can lead to increased plasma levels and potential toxicity [78]. The metabolism of warfarin is also altered, leading to changes in its anticoagulant activity [79]. These drugs are frequently used by older patients, including those reliant on the care of others [80]. However, liver senescence is particularly relevant with drugs that have a narrow therapeutic index (NTI drugs) or are primarily metabolized by the liver [81]. In that manner, the path for senescence is paved in cases of medical negligence. If a healthcare professional fails to account for changes in the liver due to senescence when prescribing or monitoring medications in older patients, and this leads to harm, it could form the basis of a liability claim [82,83,84].

Given these aspects, clinicians may need to adjust pharmaceutical dosages for patients with documented hepatocyte senescence or hepatocyte SASPs. This could help minimize toxicity and ensure efficacy. In addition, it is useful for clinicians to consider alternative medications less impacted by senescent hepatocyte changes. The close monitoring of drug levels, liver function, and potential toxicity is crucial for patients with aging hepatocytes.

### Substance Abuse

In toxicology cases, impaired metabolism linked to liver senescence can lead to altered concentrations of “parent” drugs (toxins) or metabolites [85]. Additionally, liver senescence can lead to impaired ratios of these compounds. These changes can complicate the interpretation of post-mortem drug levels and the assessment of drug influence or toxicity, indicating functional decline and increased vulnerability. Overall, liver pathology documented in medical history can be linked to significant liver senescence, which is vital for accurate toxicological interpretation [3,86].

When interpreting toxicology, substance abuse is a significant medico-legal issue that can be influenced by the “zombie-like” state of the patient’s/ user’s liver or by the factors that this type of liver secretes. In senescent hepatocytes, the cytochrome enzymes (particularly cytochrome P450 (CYP450)) are downregulated [87,88], resulting in impaired drug metabolism and detoxification.

In instances of livers affected with senescence, chronic alcohol consumption can further induce hepatocyte SASPs, leading to a range of adverse effects on liver health and beyond [89,90]. For instance, alcohol-triggered hepatocyte SASPs can cause increased inflammation, promoting liver fibrosis [91]. The metabolism of alcohol by hepatocytes can produce ROS, which may induce SASPs [53]. Likewise, nicotine exposure can induce epigenetic changes, oxidative stress, and SASPs in hepatocytes, producing inflammatory cytokines [92]. This can contribute to the development of liver diseases, including liver fibrosis, hepatocellular carcinoma, and MASLD [93].

However, cannabis use has also been associated with various liver-related effects, including liver damage, inflammation, and fibrosis, particularly with heavy or chronic use [94]. In large epidemiological studies, cannabis use has been repeatedly linked to liver pathologies. Still, these studies were retrospective and uncontrolled, particularly for the presence of other causes of chronic liver injury [95]. Hepatocytes express cannabinoid receptors, including CB1 and CB2, which can be activated by the cannabinoids found in cannabis [96]. Their role in liver fibrosis is anything but universal. Some studies suggest that cannabinoids may possess antifibrotic effects and reduce liver fibrosis or even mitigate hepatocyte senescence [96]. However, initial concerns regarding the effects of cannabis on the liver arose from a study in the early seventies that identified abundant liver pathology in young cannabis users. This was, however, amplified by the co-ingestion of alcohol. Nonetheless, cannabinoids may possess therapeutic potential as senolytic agents, as evidenced by their aforementioned antifibrotic potential [96].

Hallucinogens, a class of psychoactive substances, have been studied for their potential effects on hepatocyte senescence. Psilocybin, the active compound in psychedelic mushrooms, has been shown to increase cellular senescence in hepatocytes, likely due to its capacity to induce oxidative stress. Lysergic acid diethylamide (LSD) has also been found to induce senescence in hepatocytes, possibly by activating the p53/p21 pathway.

N,N-Dimethyltryptamine (DMT), a hallucinogenic compound found in various plants, has been demonstrated to induce an increase in senescence-associated β-galactosidase (SA-β-Gal) activity in hepatocytes [97]. Ayahuasca, a plant-based psychedelic brew, has also been found to induce senescence in hepatocytes, likely due to its ability to trigger oxidative stress and activate the p53/p21 pathway [98]. LSD produced higher scores on the Five-Dimensional Altered States of Consciousness (5D-ASC) Rating Scale than did psilocybin or DMT [99].

The chronic use of hallucinogens may accelerate aging and elevate the risk of age-related diseases, including liver disease [100]. The senescence of hepatocytes induced by hallucinogens may contribute to liver toxicity and dysfunction [14,101].

Despite limited research on new psychoactive substances (NPSs), these substances may contribute to hepatocyte aging through various mechanisms. NPSs may precipitate oxidative stress, mitochondrial dysfunction, and inflammatory responses. The interaction between NPSs and SASPs in hepatocytes and NPS-induced liver damage underscores the necessity for further investigations into the underlying mechanisms [102,103,104].

In the same vein, various liver-related effects have been assigned to cocaine use, including hepatocyte senescence resulting from oxidative stress, inflammation, or apoptosis. Likewise, cocaine can cause liver injury via conversion into a toxic metabolite during metabolism [105]. Cocaine users may be more susceptible to liver injury from other sources, such as viral hepatitis or alcohol use. Additionally, there have been documented cases of heroin-induced pulmonary edema, acute cardiac injury, and acute rhabdomyolysis in cocaine users [106,107]. This can lead to impaired liver function and an increased risk of liver disease. Cocaine-induced hepatocyte senescence can lead to impaired liver function, including reduced detoxification capacity and altered glucose metabolism [106].

Opiate use can promote hepatocyte senescence through various mechanisms, including cellular stress, DNA damage, and epigenetic changes. This can lead to impaired liver function and an increased risk of liver disease [107]. Opioids are an uncommon cause of drug-induced liver disease and are not mentioned in major case series of clinically apparent liver injury [108].

## 6. Supporting Evidence-Based Expertise

Testing for hepatocyte SASPs can be challenging, regardless of the motivation of the evidence-based expertise. Among several methods that can help in detecting and monitor senescence in hepatocytes, histological examination for signs, such as senescent hepatocytes and inflammatory infiltrates, is the only truly objective method. Various imaging techniques, such as magnetic resonance imaging (MRI) and positron emission tomography (PET) scans, can detect changes in the liver associated with senescence. Likewise, a wide range of molecular methods, including serum levels of SASP-associated cytokines and growth factors, can be used to detect and monitor signs of senescence in hepatocytes. There are several in vitro methods, such as cellular culturing, senescence-associated beta-galactosidase (SA-β-Gal) staining, P16 and P21 immunostaining, enzyme-linked immunosorbent assay (ELISA), and multiplex assays.

### Molecular Testing

**Figure 2:**
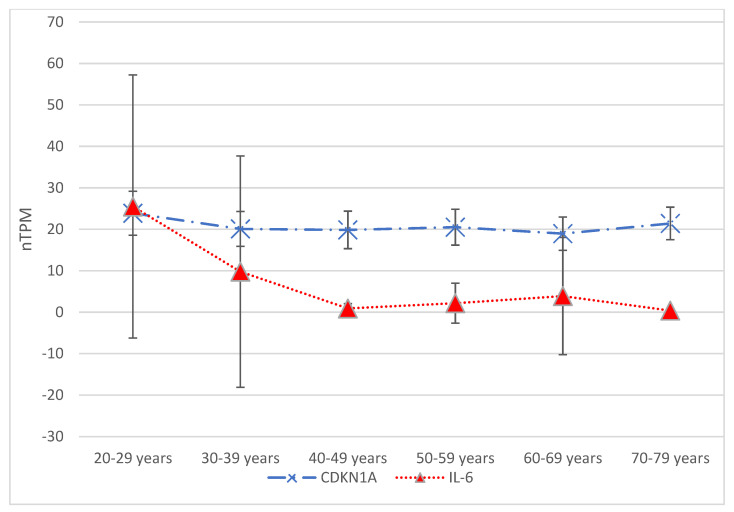
Gene expression data from The Human Protein Atlas© (available at https://www.proteinatlas.org/, accessed on 15 April 2025) are presented in terms of transcripts per million (nTPM) across six age groups: 20–29 years, 30–39 years, 40–49 years, 50–59 years, 60–69 years, and 70–79 years. Focusing on hepatocyte gene expression, two genes traditionally associated with senescence—CDKN1A and IL-6—showed significant differences in expression between the various age groups. The Tukey–Kramer test indicated a significant difference (*p* = 0.04) when comparing CDKN1A expression between the 20–29 and 60–69 age groups. Significant differences were also seen when comparing the 20–29 age group with the 40–49 age group and each subsequent age group. For comparisons involving three age groups, starting with the 40–49 age group, the *p*-value for gene expression means was found to be less than 0.01. Additionally, the 70–79 age group demonstrated a significant difference (*p* = 0.012) compared to the 20–29 age group.

Molecular testing for detecting and monitoring hepatocyte senescence and hepatocyte SASPs typically involves detecting specific biomarkers and gene expression profiles [9,109,110]. It later plays a crucial role in hepatocyte senescence, and contributes to the development and maintenance of the following senescent phenotypes [67,111]:

- Cyclin-dependent kinase inhibitor2A (*CDKN2A*, p16);
- Cyclin-dependent kinase inhibitor1A (*CDKN1A*, p21);
- *IL-6* (Figure 2).

The Human Protein Atlas© provides data on CDKN1A, CDKN2A, and IL-6 expression in hepatocytes across six age groups. The Tukey–Kramer test identified significantly different means (*p =* 0.04) when comparing the 20–29 age group to the 60–69 age group concerning CDKN1A expression. Similarly, the Tukey–Kramer test showed significant differences when comparing the 20–29 age group to the 40–49 age group and each consecutive age group thereafter. For comparing three age groups, starting with the 40–49 age group, the *p*-value for means of gene expression was less than 0.01. The 70–79 age group also showed a significant difference (*p =* 0.012) from the 20–29 age group. In contrast, regarding CDKN2A expression, the Tukey–Kramer test revealed no significant differences between the means of any pair of age groups, with the *p*-value from one-way ANOVA being 0.77.

- IL-8;
- *Vascular endothelial growth factor (VEGF)*;
- *MMPs*.

Aside from the gene expression, molecular testing includes the following:

1. Analysis of microarrays of gene expression profiles to identify senescence-associated genes and pathways [112];
2. High-throughput RNA sequencing (RNA-seq) to analyze gene expression and identify senescence-associated transcripts [113];
3. DNA methylation levels in senescence-associated gene promoters, such as CDKN2A and CDKN1A [114];
4. Histone modification analysis, such as H3K9me3 and H3K27me3, in senescence-associated gene promoters [115];
5. Telomere length measurement using techniques such as quantitative PCR (qPCR) or fluorescence in situ hybridization (FISH) [116];
6. Cytokine and growth factor analysis of levels of SASP-associated cytokines and growth factors, such as IL-6, IL-8, and VEGF, using ELISA or multiplex assays [26];
7. Proteomic analysis to identify SASP-associated proteins [84];
8. Senescence-associated beta-galactosidase (SA-β-Gal) staining—a biochemical stain that detects senescent cells [117];
9. Immunostaining of p16 and p21—markers of cellular senescence [118].

## 7. Identifying a Reliable Biomarker

Identifying reliable biomarkers of liver senescence is crucial for assessing liver function [119]. However, no single marker is exclusively present in all senescent cells or absent in all non-senescent cells. Hence, the requirement of multiple markers (Table 2) complicates standardized detection and interpretation in a medico-legal setting [109]. Circulating levels of certain molecules can reflect the presence and activity of senescent cells in the liver and other tissues. These include components of the SASP, as mentioned above, as well as other factors. Research is ongoing to identify specific circulating biomarkers accurately reflecting liver senescence [110,118].

It is important to note that while several biomarkers have been identified, a single, universally accepted biomarker does not yet exist. A combination of these markers, assessed at different points (cellular, histological, and systemic), is likely needed to evaluate liver senescence comprehensively. Furthermore, the context of the liver disease or condition being studied is crucial, as the specific biomarkers involved may vary.

## 8. Limitations of Using the “Zombie-like State” of the Liver in Legal Medicine

While the concept of “zombie-like state” (specifically related to the liver) holds promise for various applications, particularly in forensic age estimation, its widespread use in legal medicine is significantly hampered by several key limitations.

One of the primary limitations is the lack of a single, universally accepted marker for cellular senescence. A limitation is the lack of strong evidence and the absence of medico-legal literature acknowledging senescence as a potentially relevant phenomenon. Cellular senescence is essential in every modern opinion-making process related to pharmaceuticals and their metabolites. Sporadic studies of medicines and drugs of abuse that may impact liver senescence (and vice versa) are retrospective and uncontrolled, especially regarding other causes of chronic liver injury. There is nothing to say about acute liver injury in light of pharmaceuticals and their uptake.

## 9. Conclusions

Exploring the interplay of pharmaceuticals and hepatocyte senescence may enhance expert opinions and clinical reasoning. For these reasons, the constant monitoring of liver function is essential. In the management of patients taking medications known to impact hepatocyte senescence, particularly those with a history of liver disease, clinicians may need to adjust dosages or consider alternative or adjunctive treatments.

Leveling up medical professionals’ knowledge of hepatocyte senescence and the hepatocyte SASP can aid in developing treatment strategies. Specific inhibitors such as senolytics may help reduce inflammation and oxidative stress. Anyone involved in the assessment of injuries or discussing their mechanisms should be aware of these agents and the impacts on senescence of drugs. It is important to note that current therapeutic interventions do not specifically target individual types of cells, which can lead to adverse effects. This therapeutic approach can affect processes such as embryogenesis and wound healing, or it may increase the risk of tumor formation. All of this can be considered in insurance compensation claims [101,109,120].

## Figures and Tables

**Table 1:** Content analysis of the included studies regarding whether their content is related to “medico-legal” or “expert witness” topics. Individual studies were screened for specific search terms (including + senescence, hepatocyte senescence, or hepatocyte SASP). Studies explicitly related to each of these topics were allocated to a specific group/column.

Type of Study	Search Term	Forensic	Medico-Legal	Expert Witness
Primary	Senescence	21		0
	Human/non-human	5	16	
	Hepatocyte senescence	2	2	
	Hepatocyte SASP	0	0	
Secondary	Senescence	121	121	
	Hepatocyte senescence	7	1	
	Hepatocyte SASP	0	0	

**Table 2:** Overview of biomarkers of cellular senescence with a focus on the liver.

**1. Cellular and Molecular Markers**	
Cell Cycle Inhibitors	
Senescence-Associated Beta-Galactosidase (SA-\beta-gal)	
Telomere Shortening	
DNA Damage Markers	
Changes in Nuclear Morphology	
**2. Senescence-Associated Secretory Phenotype (SASP)**	
Pro-inflammatory Cytokines and Chemokines	Such as Interleukin-6 (IL-6), Interleukin-8 (IL-8), and CC motif chemokine ligand 2 (CCL2).
Growth Factors	Including growth differentiation factor 15 (GDF15).
Matrix Metalloproteinases (MMPs)	Enzymes involved extracellular matrix remodeling.
**3. Functional Biomarkers**	
**4. Imaging Biomarkers**	
**5. Humoral Biomarkers**	

ASP—senescence-associated secretory phenotype; SASP factors labeled as (2) can be measured in liver tissue, isolated liver cells, or circulation (as humoral biomarkers).
